# Sugar, Taxes, & Choice

**DOI:** 10.1002/hast.1067

**Published:** 2019-12-08

**Authors:** Carissa Véliz, Hannah Maslen, Michael Essman, Lindsey Smith Taillie, Julian Savulescu

## Abstract

*Population obesity and associated morbidities pose significant public health and economic burdens in the United Kingdom, United States, and globally. As a response, public health initiatives often seek to change individuals’ unhealthy behavior, with the dual aims of improving their health and conserving health care resources. One such initiative—taxes on sugar‐sweetened beverages—has sparked considerable ethical debate. Prominent in the debate are arguments seeking to demonstrate the supposed impermissibility of SSB taxes and similar policies on the grounds that they interfere with individuals’ freedom and autonomy. Commentators have often assumed that a policy intended to restrict or change private individuals’ consumption behavior will necessarily curtail freedom and, as a corollary, will undermine individuals’ autonomy with respect to their consumption choices. Yet this assumption involves a conceptual mistake. To address the misunderstanding, it’s necessary to attend to the differences between negative liberty, freedom of options, and autonomy. Ultimately, concerns about negative liberty, freedom, and autonomy do not provide strong grounds for opposing SSB taxes.*

## Article

Taxes on sugar‐sweetened beverages reduce consumption, but a strong public backlash holds that they compromise consumers’ liberty, freedom, and autonomy. Looking at each concept in turn—as they are interrelated and overlapping but not identical—establishes that the taxes are permissible.

Population obesity and associated morbidities pose significant public health and economic burdens in the United Kingdom, United States, and globally.^1^ As a response, public health initiatives often seek to change individuals’ unhealthy behavior, with the dual aims of improving their health and conserving health care resources. One such initiative—taxes on sugar‐sweetened beverages (or SSBs, as they are known in industry and public health parlance)—has attracted considerable attention in the media and public debate.^2^ Although taxes are effective in reducing SSB consumption,^3^ there is a wider ethical debate about the permissibility of state interference in the food and beverage market, with strong voices on both sides.

Lawrence Gostin has recently presented the case for widespread adoption of taxation strategies that would create powerful disincentives to purchase SSBs.^4^ Gostin’s claims are well‐founded: meta‐analyses have found that taxes on the beverages have been effective in reducing their consumption, and models consistently predict small reductions in average body mass index among consumers.^5^ In relation to these and similar policies, Gostin argues that the high value placed on “American individualism” over health and the “common good” plays a key role in generating public backlash against regulatory initiatives intended to promote these latter values. “Big Beverage” companies capitalize on this ranking of values, he suggests, by encouraging the perception that regulations such as taxation compromise consumer liberty and autonomy.

As Gostin notes, there is an urgent need for comprehensive scholarly and policy conversation about the regulation of sugar. As policies have been rolled out, there has been little scrutiny of their ethical permissibility, and the limited literature that exists leaves the issue unsettled. Although Gostin is clearly in favor of SSB taxation, he does not attempt an analysis of the moral arguments for and against it.

Objections to the imposition of SSB taxes and similar policies on the grounds that they interfere with individuals’ freedom and autonomy have been prominent in the public debate. When New York City mayor Michael Bloomberg proposed a ban on large sugary drinks in New York City in 2012, for example, the reaction was fierce. The *New York Times* called it “a ban too far,” and the soft drink industry caricatured the mayor as a nanny, using the headline “You Only Thought You Lived in the Land of the Free.”^6^ Similarly, in the United Kingdom, the TaxPayers’ Alliance, an activist organization, argued that “[t]he sugar tax represents an unacceptable infringement on personal liberty and freedom of choice.”^7^

Arguments seeking to demonstrate the supposed impermissibility of these policies, however, are frequently compromised by confusion regarding the concepts of freedom and autonomy. Commentators have often assumed that a policy intended to restrict or change private individuals’ consumption behavior will necessarily curtail freedom (both freedom from interference and freedom to have certain options available) and, as a corollary, will undermine individuals’ autonomy with respect to their consumption choices. Yet this assumption—that curtailment of freedom and curtailment of autonomy necessarily go hand in hand—involves a conceptual mistake. Where such an assumption is made, analysis of the permissibility of SSB beverage taxes will be inadequate.

To address the misunderstanding and gaps in the literature, it’s necessary to attend to the differences between negative liberty, freedom of options (or opportunity freedom), and autonomy. Although these three concepts are interrelated and overlapping, they require different analyses. Ultimately, we will argue that SSB taxes are ethically permissible, as concerns about negative liberty, freedom, and autonomy do not provide strong grounds for opposing them. For those who still worry about the effects on freedom and autonomy, we will suggest complementary strategies that will enable consumers to take greater responsibility for their consumption choices. We will also consider the regressive nature of SSB taxes, finding that empirical data support a net benefit to those most affected by the rising beverage costs.

## Sugar‐Sweetened Beverage Taxation in Practice

In October 2013, the Mexican government passed a law imposing a one‐peso‐per‐liter excise tax on SSBs, effectively raising their prices by about 10 percent.^8^ The Mexican sugar tax has been effective thus far in reducing purchases of SSBs, and the benefits appear to be increasing with time: compared to what would have been expected without a tax, purchases of SSBs were lower by an average of 5.5 percent in the first year of the tax and 9.7 percent in the second year.^9^ Studies suggest that the greatest reductions in SSB consumption may be achieved in the long term, as taxes and other coordinated policies modify consumer preferences and habit formation.^10^

Further evidence of the efficacy of these interventions includes a recent study of the effects of a one‐cent‐per‐ounce tax in Berkeley, California, which found that SSB consumption decreased 21 percent, while bottled water consumption increased—yet another desirable effect (for individuals’ health, if not the environment).^11^ A later study of the effects of the Berkeley soda tax after one year found mixed results: SSB sales decreased, and sales of untaxed beverages and water increased in some settings.^12^ Despite the variations in pass‐through from tax to consumer, there was no significant increase in overall consumer spending, which suggests that consumers were taking the price increase into account when making their purchases and were not being punished by spending more overall. Although SSB sales were reduced in Berkeley, purchases increased in the surrounding area, suggesting that taxes can lead to consumption increases outside the taxed jurisdiction, where they are less expensive. State‐level policies might minimize such loopholes.

In Chile, a small tax on beverages with greater than 6.25 grams of sugar per 100 milliliters led to accordingly small reductions in purchases of those beverages.^13^ Although questions still remain regarding the optimal level of taxation and the amount of reduction that is necessary to significantly improve population health, systematic reviews of the academic literature consistently find that SSB taxes are effective in reducing consumption.^14^

In the United Kingdom, the soft drinks industry levy, implemented in April 2018, established that beverages containing 5 to 8 grams of sugar per 100 milliliters are taxed at 18 pence per liter, and beverages containing more than 8 grams of sugar per 100 milliliter are taxed at 24 pence per liter.^15^ This scheme creates new opportunities for industry responses. Rather than uniformly punishing parties for the sale and consumption of SSBs by volume, the U.K. tax allows companies to reduce or altogether avoid taxation if the added sugar content is below a threshold. Such reformulation could greatly reduce the economic burden on both producer and consumer. The U.K. SSB tax can thus be expected to have a dual effect: to spur industry reformulation, thereby reducing the amount of sugar in the drink supply, and to incentivize consumers to purchase less of these beverages.

Consumer spokespeople and academic commentators have questioned whether SSB taxes have unjustifiable implications for consumer choice. Such arguments appeal to the idea that sugar taxes reduce individuals’ freedom and compromise their autonomy. If we consider that tax interventions would be successful precisely by virtue of having intentionally changed the value of the consumption choices (and associated behavior), such arguments have some prima facie appeal. It might seem that if regulators can predictably determine what you buy and consume, then your choices are not completely your own—an idea that beverage companies have used to stir up public discontent with regulatory measures. Whether this regulatory mechanism compromises consumer freedom or autonomy is precisely the question that concerns us.

In what follows, we first provide definitions of the key concepts of negative liberty, freedom of options, and autonomy, demonstrating that they are not uniformly compromised by state regulation. We then provide some examples of conceptual imprecision in the literature. Finally, we evaluate the permissibility of SSB taxes, employing our conceptual distinctions.

## Negative Liberty, Freedom of Options, and Autonomy

Most philosophers agree that the concepts of freedom and autonomy differ in their detail and should be distinguished. “Negative liberty” refers to “freedom from” external influences, particularly those intended to control or restrict one’s actions.^16^ In Amartya Sen’s language, negative liberty consists of “immunity from encroachment.”^17^ Negative liberty is often at stake in discussions of the proper limits of state interference, with state paternalism being seen as inimical to negative liberty. Banning smoking, for example, is a restriction of negative liberty. Restricting this form of liberty is most easily justified when doing so prevents the agent whose negative liberty is contravened from causing harm to others, in line with John Stuart Mill’s harm principle, which holds that power can be rightfully exercised over members of society against their will only for the purpose of preventing harm to others.

“Freedom of options,” or “opportunity freedom,” in contrast, refers to having available opportunities to do certain things—an agent’s set of substantive opportunities. Isaiah Berlin called this “positive liberty.”^18^ This concept has been distinguished and employed in political and economic theory, particularly in the context of consumer choices. This positive dimension of freedom, which Sen calls “opportunity freedom,”^19^ relates to the agent’s “opportunity to achieve.”^20^ To enjoy freedom of options, it is necessary to have access to a reasonable range of options; one option is never enough. It is likewise important that the options in question be adequate (in other words, an agent who has access to three good options can be said to enjoy more freedom of options than one who has access to three bad options).

In the broadly procedural sense most commonly invoked in discussions of paternalism, “autonomy” refers to making choices in accordance with one’s long‐term goals and values. The degree to which a certain act has been performed or made autonomously will depend on the comprehensiveness of the factual information available to the agent (on the basis of which she makes her decision); the extent to which the agent has considered, imagined, and evaluated the implications of the act; and the accessible alternatives.^21^

While these terms are conceptually distinct, they are also interrelated. Autonomy, for instance, is commonly understood to require not simply that the agent make her own decisions but also that she have the negative freedom to act on those decisions. Similarly, some theorists claim that, to make autonomous decisions, one must first have a sufficient degree of freedom of options,^22^ such that one’s choices are not a reflection of adaptive preference formation (the unintended altering of our preferences in light of the options available). Nonetheless, distinguishing between negative liberty, freedom of options, and autonomy is helpful to assess the effects of SSB taxes, diagnose problems, and identify solutions more precisely. In objecting to so‐called sin taxes, critics have often cited freedom and autonomy. When these concepts are used interchangeably, assessing the strength of the arguments they are intended to support becomes difficult. Sugar taxes may not affect all types of freedom and agents’ prospects for autonomous decision‐making to the same extent and with the same implications.

***Less freedom does not necessarily entail less autonomy.*** Reductions of either negative liberty or freedom of options do not necessarily compromise autonomy. A state intervention banning smoking might, in some cases, promote an agent’s autonomy, even when the agent would have smoked had the ban not been in place. If the agent has a rationally endorsed preference to stop smoking but finds it difficult to resist temptation, a ban on the sale of cigarettes might promote her autonomy by making it much harder for her to deviate from her valued goal of not smoking. This is so even though her negative freedom is compromised by state interference in her liberty. Indeed, one way to be more autonomous is through Ulysses‐style precommitment contracts, which limit one’s future options in order to support one’s values in the face of temptation or distraction.^23^ Thus, reductions in negative liberty need not have a detrimental effect on autonomy. When such reductions do impinge on citizens’ autonomy (when, for example, the state forbids citizens from physically assaulting others), state interferences must be judged according to their own merit, recognizing that state encroachment can sometimes be justified. Preventing harm to others and promoting justice are two considerations that can justify restricting agents’ autonomy.

Similarly, reducing freedom of options will not necessarily reduce an agent’s autonomy, especially when the agent does not value the options that are removed (or at least values other options equally or more).^24^ If the state creates a law prohibiting people with eyesight of less than a certain quality from being airline pilots, and an agent does not meet the threshold, this does not necessarily mean that her choice of an alternative career as a doctor is less autonomous than it would have been had she had the option open to be an airline pilot. Perhaps she never wanted to be a pilot, or she wanted to be a doctor just as much as she wanted to be a pilot.

***More freedom does not necessarily entail more autonomy.*** It is also true that increases in an agent’s negative liberty or freedom of options—reducing state interference or increasing opportunities—do not necessarily increase autonomy. As Gerald Dworkin argued, more choice is not necessarily better for autonomy.^25^ Suppose that, alongside the familiar brand of biscuits you are used to buying in the supermarket, a hundred other, less heavily state‐taxed brands are added to the shelves. And suppose you still buy the biscuits you bought before, more out of habit than as a result of thorough research into the hundred new brands. The new options have only made it harder for you to find the brand you know. That an agent is free from external influence does not mean that she necessarily acts autonomously (in other words, she can act out of habit, impulsively, or unreflectively), and increasing options or reducing state interference does not directly render the agent more autonomous. In fact, too many options can decrease autonomous decision‐making by overwhelming the agent—the so‐called paradox of choice.^26^

In addition to the role of factual information in making autonomous choices, we should also consider the psychological obstacles to autonomous consumption, which will persist regardless of the number and range of options available to the consumer. For example, relevant to sugar‐sweetened beverages, evidence suggests that the added calories someone consumes from such beverages do not lead to decreases in the person’s food consumption.^27^ This phenomenon can be seen as a type of information gap between the calories and the consumer: sugary beverages do not provide satiety feedback to the brain, which would halt consumption of other calories. Further obstacles to autonomous consumption may be generated by food marketing, which might cause consumers to be more likely to act in a way that makes it hard to respond to natural satiety cues. For example, the expectation of drinking an “indulgent” versus a “sensible” shake differentially affects the release of the hunger‐regulating hormone ghrelin.^28^

In summary, a person makes an autonomous choice when they do so reflectively and in line with their deeply held goals and values. While autonomy requires *sufficient* negative liberty and freedom of options, the extent to which the agent acts autonomously does not necessarily track their degree of negative and opportunity freedom. Rational endorsement of one’s choices is necessary for autonomy but not for freedom, and increased freedom is not sufficient for autonomy. Thus, restrictions on freedom do not necessarily diminish autonomy.

Unfortunately, the existing literature on SSB taxation policy does not always keep these distinctions clear. Consider the following passage by Rebecca Green, in which negative liberty, freedom of options, and autonomy are all invoked without distinction:

Sin taxes may be opposed … if framed within a libertarian rights perspective. Brannigan and Boss (2001) characterized libertarians as being concerned with personal autonomy over all other rights and obligations…. It is a critical point that while sin taxes may create a negative, inhibitory consequence to a harmful behavior, they do not, in fact, prohibit or criminalize the behavior. The individual remains autonomous in having the behavior as an option; the tax is simply another factor (along with potential negative health consequences) to consider when engaging in the behavior.^29^

Green first invokes libertarianism, implying that interference from the state is incompatible with personal autonomy. She then seems to suggest that retaining options (opportunity freedom) is sufficient for autonomous decision‐making.

Assumptions about a necessary relationship between types of freedom and autonomy are also present in these remarks by Jonathan Cummings:

This principle of liberty from government intervention pervades to this day, and the historical, cultural, and traditional respect for autonomy is one of the rationales for opposing paternalistic policies like sin taxes…. [P]olicies that infringe on liberty do not respect humans as autonomous moral agents—humans as “ends in themselves”—and therefore, these policies should not be implemented.^30^

Cummings equates reductions in negative liberty with compromised autonomy, also suggesting that any such policy treats individuals merely as means. While the concepts invoked by these and other authors are indeed relevant to assessing the permissibility of policies such as those establishing SSB taxes, the question is more adequately addressed when freedom and autonomy (and their interactions) feature as distinct but interrelated considerations.

Some authors have made significant progress in unraveling the complicated relationship between freedom and autonomy in the debate on taxation of unhealthy products. For example, Anne Barnhill and Katherine Francis King argue that there are multiple “dimensions of autonomy”: freedom from external constraints, psychological capacity to make choices, and understanding available options.^31^ They contend that the value of autonomy motivates both normative reasons *against* taxation policies, when those policies compromise autonomy along one dimension, and normative reasons *for* such policies, when those policies strengthen autonomy along another dimension. Although they do not develop their argument further, their parsing out of different elements of autonomy is a good example of the level of nuance and complexity that is necessary for an adequate ethical analysis of sugar taxes.

In the remainder of this article, we will extend this debate through further close examination of the concepts of negative liberty, freedom of options, and autonomy. Doing so is important not only to advance the academic debate but also to formulate principled rebuttals to the emotive lobbying efforts of the Big Beverage industry and other stakeholders. We examine whether SSB taxes are consistent with enough freedom from external influence and allow enough freedom of options to preserve the possibility for autonomous choice. We draw on empirical evidence from existing taxation schemes to examine whether and how these dimensions of interest may be compromised or even promoted by SSB taxes.

## Effects on Citizens’ Freedoms and Autonomy

When commentators object that taxes on unhealthy products reduce agents’ freedom and autonomy, are they correct? And if the taxes have that effect, are they impermissible?

***Negative liberty: Does an SSB tax constitute unreasonable state interference?*** The concern that an SSB tax might compromise negative liberty focuses on the question of whether the imposition of such a tax constitutes unjustified interference in the lives of citizens. Following philosophers such as John Locke and Robert Nozick, libertarians who hold a particularly conservative view on the limits of the state could argue that it should not be permitted to interfere in the transfer of legitimately acquired holdings from one individual to another—in other words, that the market must always be free, unless intervention is required to protect against or rectify rights violations. On such a view, companies should be free to set the terms for the transfer of their goods, a significant aspect of which is setting prices.

This encroachment, however, principally affects the negative liberty of the sellers, leaving the purchasers, with whom ethical discussions tend to be most concerned, unaffected. Interfering in the seller’s freedom to set the terms of the transfer of their goods does not constitute encroachment into the negative rights of the buyer because it is sellers who set prices. Sellers are free to assume the cost of the tax by lowering the prices of their products. Although the libertarian might argue that the state’s interference in the free market constitutes a procedural encroachment, the impermissibility of which is independent of the particular effects on individuals, the negative liberty of the buyer is not the principal casualty of such a tax. Indeed, following such thought, libertarians would equally object to state‐enforced *subsidization* of prices. Thus, the libertarian concern with negative liberty would amount to a concern with the freedom of the market. Given that all societies accept some degree of state interference in the market (for example, other kinds of taxes such as sales tax, food safety regulations, and so on), the burden would be on detractors to show why an SSB tax would be unduly onerous. If we think other kinds of taxes are justified by virtue of having some kind of common good or public health justification and are not so burdensome as to altogether prohibit an activity that citizens value (such as drinking sugary drinks), then critics must show why SSBs taxes are any different. In other words, sugar taxes are on equal footing with other taxes in that they have a public health justification and are not a ban. Therefore, the burden of proof is on those who want to argue that *all* taxes are unduly onerous or those who can show that there is something about sugar taxes that makes them more onerous than other taxes.

When writers object to SSB and other taxes on the grounds that they represent unreasonable state interference, arguments usually invoke the language of paternalism. It could be argued that taxes that cover the negative externalities (costs suffered by society) of consumption are acceptable, whereas paternalistic taxes designed to decrease consumption constitute an unjustified interference in the market. But while this distinction might be relevant in other cases, we do not have to settle this debate for the case of SSB taxes, as sugar consumption does create health costs, and these taxes will typically help the state cover those costs. Whether there is a paternalistic motivation behind the tax is, for the purposes of our discussion, beyond the point.

Even if one were persuaded that SSB taxes fall principally on the shoulders of sellers, and the revenue were used to cover negative externalities, one could still think that such taxes infringe on the negative liberty of individuals. For example, one might try to argue that if an individual wants to buy a sweetened beverage for $1.00, a company wants to sell the product for that price, but if an SSB tax makes that drink cost $1.25, then a law preventing sale at the original price infringes on individuals’ liberty. Taxes like that of the United Kingdom, however, do not impose a particular price on any given beverage but, rather, charge a certain amount per liter for drinks that contain more than an established threshold of sugar. If a company still wants to sell their product at $1.00, then they can lower the price of the drink before taxes (and earn less from every sale), they can sell smaller portions, or they can alter the recipe so that it contains little enough sugar to avoid the tax. In other words, the onus is principally put on companies to cater to customers’ desires while respecting the law.

***Freedom of options: Do SSB taxes reduce the options available to the agent?*** “Freedom of options” refers to the options open to the agent. Whether SSB taxes reduce individuals’ freedom of options will depend on how we specify the options available in their option set and whether a given tax removes more meaningful options than it adds. SSB taxes will remove the option of purchasing a slightly cheaper beverage than would have been available absent the tax. Assuming most agents prefer to pay less rather than more for goods, the remaining option to purchase the slightly higher‐priced good is rendered to some small degree less desirable to the agent. A tax changes the desirability or value of the beverage, but it does not remove it as an option.

Furthermore, an SSB tax might result in institutions’ incentivizing the consumption of other, alternative products or of different portions of the same beverages. Under the Mexican scheme, part of the money raised by the tax was intended to subsidize attempts to increase the availability of clean, potable water, adding a further option to the option set (or, at least, making this option easier to obtain).^32^ In the United Kingdom, Coca‐Cola shrank 1.75 liter Cokes to 1.5 liters in response to the sugar tax.^33^ Such concurrent effects alter the relative desirability of options available to the consumer, with the possibility that the overall option set remains equally or even more desirable to the consumer, depending on her personal preferences.

If the sugar tax makes a product expensive enough, then there may be cases in which the option of purchasing SSBs in the same quantity as without the tax is effectively removed. In the United Kingdom, the price of a 500 milliliter bottle increased 25 percent.^34^ If individuals can no longer afford to purchase SSBs in the same quantities as they used to, then the option to do so has not just been made less desirable relative to the untaxed state—it is no longer available. In such cases, the question, as it pertains to freedom of options, is whether the remaining option set comprises a *sufficient* number of real options. As mentioned, according to some philosophers, such as Joseph Raz, having a range of adequate options available is a prerequisite for autonomy.

Clearly, it is not the case that autonomy (or justice) requires states to ensure that all agents have *all* options open to them. It is enough for agents to have *enough* options. If, as consistent with the Mexican policy, clean, potable water is made more easily available through the revenue generated by the tax, then enough options remain. Of course, the precise point at which the number of options is sufficient is bound to be controversial. But the United Kingdom still has dozens of options, and that seems uncontroversially sufficient. Even if the removal of the option to drink SSBs in the quantity one did absent the tax is not coupled with an increase in another option, it would seem implausible to claim that the options that remain are insufficient to allow for autonomous choice. Most choices regarding beverages, including choices that are not necessarily healthier, remain untouched by an SSB tax: people can still make lemonade and tea at home, buy sparkling water or fruit juices with no added sugar, or purchase drinks made with artificial sweeteners, among other options.

Moving beyond the question of the effects on the options pertaining to beverages, how might such sugar taxes affect an individuals’ freedom of opportunity more generally? Given that many aspects of good health are instrumental to fully enjoying freedom of options, the consumption of SSB itself can alter an agent’s opportunity freedom via the effects that high consumption can have on health. Sugar consumption is associated with risk of dental caries,^35^ which typically cause pain and can impair one’s ability to enjoy a range of foods. High consumption of SSBs is also associated with increased risk of the metabolic syndrome and type 2 diabetes.^36^ Diabetes contributes a high burden of death and disability globally.^37^ Health problems associated with diets high in sugar will often restrict an agent both physically and emotionally (for instance, there is a strong link between diabetes and depression).^38^ Thus, even if SSB taxes were to reduce individuals’ freedom of options in available beverages, the restriction might lessen the chance that their opportunity freedom to pursue other, perhaps more valuable undertakings will be limited by the health impairments that overconsumption of SSBs can lead to. In Mexico, it has been estimated that the tax will result in about 189,300 fewer cases of type 2 diabetes, 20,400 fewer strokes, and 18,900 fewer deaths occurring from 2013 to 2022.^39^

In pointing to the prospect that good health may compensate for reductions in beverage options, however, we are not assuming an ordering of the *value* of freedoms that may be incongruent with the ordering held by many agents. In contrast to our perspective, some philosophers claim that, to be rational, agents must value health over behavior that leads to ill health.^40^ Others, however, have persuasively argued that we should not assume a particular weighting of values, either as a contingent fact or as a matter of rationality.^41^ Agents might rationally decide that consuming beverages that impair their health is valuable enough to them to outweigh the disvalue accrued from that impairment.^42^ Agents trade off health for other goods all the time—when they drink alcohol, engage in risky sports, or use their time to do things other than get sufficient exercise. It should not be assumed that such a weighting of goods is irrational.

So far, we have argued that negative liberty is not unreasonably compromised, except perhaps in the most extreme libertarian accounts. Similarly, the agent’s freedom of options is either not reduced (because new beverage options become available) or is more complicatedly rebalanced through the gain in (or protection of) options generated by the absence of SSB‐related health impairments. Whether this rebalancing is problematic is best answered through an evaluation of the implications it has on the ability of agents to be self‐governing.

***Autonomous choices: Do SSB taxes undermine consumers’ autonomy?*** Even though having an adequate number of real options is necessary (but not sufficient) for autonomy, autonomy does not require that *every* option be available to the agent.^43^ In the context of a sufficient option set, making an autonomous choice requires reflection on and evaluation of alternatives, freedom from third‐party manipulation, and, ideally, coherence with one’s other rationally endorsed goals and values.

SSB taxes have been successful in reducing demand. Given the above condition of freedom from third‐party manipulation, it is reasonable to ask whether SSB tax policies have manipulated consumer choices in a way that undermines autonomy. We contend that this is not the case. Agents can make fully autonomous choices in the context of reduced options or changes in incentives. Agents’ choices before a shift in the price of a product will already be sensitive to the preceding incentive structure: price will always be a consideration when deliberating whether to purchase a food or drink—it is not as if the pretax price did *not* have an influence on the decision to purchase. The pretax situation is not a neutral one in which individuals are free from external influence. Demand is always sensitive to price, not only when prices increase. Thus, the pre‐ and posttax situations are on equal standing regarding their influence on autonomy.

Plenty of evidence supports the claim that, regardless of the lack of tax, consumers are regularly subjected to misinformation and manipulation, ranging from price setting to more subtle methods such as illuminating products to make them appear more desirable.^44^ Companies and food retailers use marketing strategies to influence the consumer, including product placement, creative packaging, price setting, price promotions, placement within stores, health messaging (often misleading), and marketing and promotions targeting children (whose preferences will track into adulthood).^45^ The agents’ default setting is not one of purely unrestricted free choice devoid of external interference, but of responding to explicit third‐party influence and even manipulation through marketing. Public health policy measures are another addition to the mix, but their intent is to promote healthier choices. Selling a product is not an inherently freer enterprise than promoting health through public policy, and it seems unfair to demand more restrictions from the state than from businesses when the state’s objectives reflect a concern for the well‐being of its citizens. The state should not be held to greater restraint than the SSB beverage industry, whose objective is to sell as many beverages as possible, even if those drinks have been demonstrated to be detrimental to its customers’ health.^46^

Another reason that an SSB tax would not influence autonomy negatively is that a change in an option’s price can prompt reconsideration of the option’s value. If an agent who habitually purchases a sugar‐sweetened beverage is confronted with a small price increase, this may prompt her to consider more alternatives and to reflect on the value she derives from the beverage. Except in cases of poverty, she is not prohibited from sticking with her default choice. If she continues with it after reflecting on the value that she places on it, then she may even be purchasing more autonomously, depending on the content and depth of her reflection. If she considers and decides to purchase an alternative, this deliberation also serves her autonomy. At the very least, it does not compromise it.

## Responsibility and Justice

We have sought to show that SSB taxes do not undermine agents’ autonomy in making decisions between a sufficient set of beverage options. Furthermore, health departments could complement taxation policies meant to reduce SSB consumption with initiatives designed to help individuals make more autonomous consumption decisions and take more responsibility for their overall health.

For example, an SSB tax could be complemented by initiatives to increase autonomous choices with respect to beverage consumption (such as information campaigns or regulation of marketing). One key country that will be evaluated for the impact of comprehensive policy approaches to obesity prevention is Chile. Recent laws that have been passed in Chile concern improved front‐of‐package labeling, marketing restrictions on products targeting children, and sugary beverage taxes.^47^ Nudging policies can also influence consumers toward healthier choices, and subsidizing healthy foods might also be a good idea. Although taxes can be part of a state effort to improve the food environment, they are not the only possible solution.

One drawback to SSB taxes is that they disproportionately burden lower‐income populations. This problem can be offset, however. Given that the poor have the greatest burden of chronic disease associated with poor diet, they stand to gain the most from public health policies to improve diets.^48^ A recent modeling study from Australia sought to quantify this claim and found that the two lowest income quintiles would experience half of the total health benefit of the tax.^49^ Although some evidence seems to suggest that low‐income consumers reduce their purchases more than other groups in response to SSB taxes,^50^ a systematic review of the health effects also found that these concerns about regressivity may be overblown, with small differences in total financial burden between income groups.^51^ On balance, the net effect of the tax on low‐income groups is positive, particularly if revenues are earmarked to fund progressive initiatives and transfers of wealth in which the money taken from low‐income groups can be returned to them.

In any event, the objection that SSB taxes are inherently regressive is distinct from objections pertaining to freedom and autonomy. With respect to those concerns, SSB taxes do not represent a threat for citizens, and they can be an important component of public health policies meant to promote health, well‐being, and personal responsibility.

## Acknowledgments

This work is supported by the Wellcome Trust through grants WT104848/Z/14/Z and 203132/Z/16/Z.
